# Boosting CO_2_ adsorption and selectivity in metal–organic frameworks of MIL-96(Al) *via* second metal Ca coordination†

**DOI:** 10.1039/d0ra00305k

**Published:** 2020-02-25

**Authors:** Hussein Rasool Abid, Zana Hassan Rada, Yuan Li, Hussein A. Mohammed, Yuan Wang, Shaobin Wang, Hamidreza Arandiyan, Xiaoyao Tan, Shaomin Liu

**Affiliations:** WA School of Mines: Minerals, Energy and Chemical Engineering, Curtin University WA 6102 Australia hussein.abid@curtin.edu.au Shaomin.Liu@curtin.edu.au; Environmental Department, Applied Medical Science, University of Karbala Karbala 56001 Iraq; Department of Chemical Engineering, Tianjin Polytechnic University Tianjin 300387 China liyuan@tjpu.edu.cn; School of Chemistry, Faculty of Science, The University of New South Wales Sydney New South Wales 2052 Australia; School of Chemical Engineering, University of Adelaide SA 5005 Australia; Laboratory of Advanced Catalysis for Sustainability, School of Chemistry, The University of Sydney Sydney 2006 Australia

## Abstract

Aluminum trimesate-based MOF (MIL-96-(Al)) has attracted intense attention due to its high chemical stability and strong CO_2_ adsorption capacity. In this study, CO_2_ capture and selectivity of MIL-96-Al was further improved by the coordination of the second metal Ca. To this end, a series of MIL-96(Al)–Ca were hydrothermally synthesised by a one-pot method, varying the molar ratio of Ca^2+^/Al^3+^. It is shown that the variation of Ca^2+^/Al^3+^ ratio results in significant changes in crystal shape and size. The shape varies from the hexagonal rods capped in the ends by a hexagonal pyramid in MIL-96(Al) without Ca to the thin hexagonal disks in MIL-96(Al)–Ca4 (the highest Ca content). Adsorption studies reveal that the CO_2_ adsorption on MIL-96(Al)–Ca1 and MIL-96(Al)–Ca2 at pressures up to 950 kPa is vastly improved due to the enhanced pore volumes compared to MIL-96(Al). The CO_2_ uptake on these materials measured in the above sequence is 10.22, 9.38 and 8.09 mmol g^−1^, respectively. However, the CO_2_ uptake reduces to 5.26 mmol g^−1^ on MIL-96(Al)–Ca4. Compared with MIL-96(Al)–Ca1, the N_2_ adsorption in MIL-96(Al)–Ca4 is significantly reduced by 90% at similar operational conditions. At 100 and 28.8 kPa, the selectivity of MIL-96(Al)–Ca4 to CO_2_/N_2_ reaches up to 67 and 841.42, respectively, which is equivalent to 5 and 26 times the selectivity of MIL-96(Al). The present findings highlight that MIL-96(Al) with second metal Ca coordination is a potential candidate as an alternative CO_2_ adsorbent for practical applications.

## Abstract 2

MIL-96(Al)–Ca1 shows the highest CO_2_ adsorption capacity; while MIL-96(Al)–Ca4 displays a distinguished morphology with the highest selectivity of CO_2_/N_2_.

## Graphical abstract

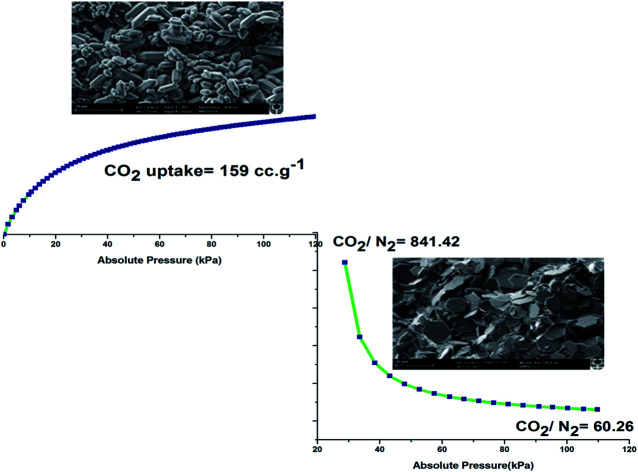

## 1. Introduction

Global warming is considered a serious disaster facing our planet. MOFs have been studied extensively for capturing greenhouse gases from gaseous mixtures.^1^ Capture of these gases by adsorption techniques is practically used in industries. Conventional porous materials such as activated carbon,^2^ zeolites^3^ and metal oxides^4^ are reliable adsorbents to control emission of greenhouse gases into the atmosphere. Recently, metal–organic frameworks (MOFs) have been a hot topic due to their great potential in different industrial applications^5^ including greenhouse gas storage and separation.^6,7^ In comparison with conventional materials, these MOF materials have a limited thermal and chemical stability; but possess larger surface areas with tunable pore sizes and pore volumes and facile functionalisation, making them attractive alternative adsorbents to be applied in various environmental fields. MOFs are synthesised by coordinating multidentate organic linkers with transition metal ions (or their clusters) into periodic porous frameworks.^8^ Popularly, single metal–organic frameworks are based on a transition metal such as Al^3+^,^9^ Fe^3+^,^10^ Cr^3+^,^11^ V^3+^,^12^ Zn^2+^,^13,14^ Mg^2+^,^15^ Mn^2+^,^16^ Co^3+^,^7^ Cu^2+^,^17^ Zr^4+^ ^18^ or Ti^4+^.^19^ MOFs are distinguished as porous materials, which can be easily modified to suit various applications.^20–22^ One of these important modifications is to synthesise MOFs with a diversity of metals accommodated in their structure.^23^ This modification can be accomplished using two methods called direct synthesis modification (DSM) and post synthesis modification (PSM) for the synthesis of mixed metal–organic frameworks (M-MOFs).^24^

DSM aims to enhance the textural characteristics and active functionalities of the parent MOFs by adding the second metal and other chemicals in one pot during the synthetic process.^25^ However, DSM is very limited in preparation of M-MOFs because of the unfavourable existence of the central and second metal in the same reaction pot, which may result in unstable frameworks with weak topologies and functional groups.^26^ To overcome this problem, suitable metals of similar ionic radii and coordination geometry are considered to maintain the integrity of the structures in M-MOFs during the DSM synthesis. On the other hand, PSM is mostly used in the preparation of M-MOFs for different applications.^27^ This modification can be achieved by controlling the activation procedure, which can build M-MOFs with well-constructed features.^28^ The main limitation of this method lies in unmatched physical or chemical properties between the main and the second metals.^29^ Furthermore, due to the small aperture size and the high steric hindrance within the structures of MOFs, it is difficult to achieve the desirable metal molar ratios by PSM or to attain a uniform distribution of the two metal ions in the final M-MOF, which may lead to undesirable alternation or collapse of the final M-MOF structure.^30^ Thus DSM can be more reliable to synthesise M-MOF if the selection of a second metal is carefully controlled according to the research target. Likewise, it has been confirmed that the preparation of M-MOFs by using incompatible metals in the direct synthesis results in the formation of a similar structure of the parent MOFs with a very low content of the second metal, but the textural properties and vacant metal sites are enriched.^31^ Recently, M-MOFs have been synthesised to improve the characteristics of MOFs for use in specific applications such as catalysis,^32^ sensing, illumines,^33^ and gas storage.^34^ In addition, CO_2_ has been a major component of greenhouse gases that have been hugely emitted from fossil fuel-fired power plants.^35^ CO_2_ can be effectively adsorbed and separated from other gas (such as N_2_) by MOFs or M-MOFs with high selectivity.^36^ In particular, the direct synthesis of M-MOFs leads to an increase in the concentration of open metal sites or defects to further increase the CO_2_ uptake and selectivity.^37^ Furthermore, the selectivity of CO_2_/N_2_ is also governed by textural properties and functionalities of MOFs.^38^ Nowadays, many researchers attempt to modify the recently developed MOFs to enhance the storage capacity of CO_2_ and simultaneously increase its separation factor from other gases.^39^ However, there are few studies dedicated to using the second metal to modify MOFs to improve their CO_2_ adsorption. This is the major motivation of the present study. Recently, a microporous Al trimesate-based MOF, denoted MIL-96-(Al), has attracted intense attention because of its high hydrothermal stability and strong CO_2_ adsorption capacity caused by the good affinity for CO_2_ due to the presence of Al Lewis acid sites and bridging –OH groups in the framework.^40^

In this work, we further improved the performance of MIL-96(Al) for CO_2_ uptake with enhanced selectivity of CO_2_/N_2_ by modifying its textural properties *via* the introduction of the incompatible metal Ca into the framework. For this purpose, DSM was applied to synthesise MIL-96(Al)–Ca samples using Al^3+^ as the main metal and Ca^2+^ as the second one. A series of MIL-96(Al)–Ca samples with different ratios of Ca^2+^/Al^3+^ were synthesised and characterised. After Ca coordination, the MIL-96(Al)–Ca samples were activated by methanol to exchange most coordinated Ca^2+^ and to enhance the concentration of vacant metal sites and defects in the molecular structure of MIL-96(Al)–Ca samples. Subsequently, these specially tailored bimetallic MOFs were tested for CO_2_ and N_2_ adsorptions. The present results indicate that MIL-96(Al) with a low Ca content can be used as an excellent adsorbent for capturing CO_2_; by contrast, MIL-96(Al) with high Ca content can be applied as a novel adsorbent for separating CO_2_ from N_2_.

## 2. Materials and methods

### 2.1 Materials

All chemicals were supplied from Sigma Aldrich-Australia without further purification including aluminium nitrate nonahydrate (Al(NO_3_)_3_·9H_2_O, ACS reagent, ≥98%), trimesic acid (BTC); 1,3,5-benzenetricarboxylic acid (C_9_H_6_O_6_, 95%), calcium carbonate (CaCO_3_, ≥99.0%), nitric acid (HNO_3_, 72%), hydrochloric acid (HCl, 32%), absolute methanol (CH_3_OH, 99.8%) and absolute ethanol (C_2_H_6_O, 99.8%). Deionised water was supplied from the ultra-high pure water system. Teflon-line autoclave – 4744 Acid Digestion Bomb of 125 mL was supplied by John Morris Scientific Pty Ltd-Australia.

### 2.2 Synthesis procedure

MIL-96(Al) and MIL-96(Al)–Ca*N* (*N* = 1, 2, 3, 4) were synthesised according to a previously reported typical procedure.^41^ Al (NO_3_)_3_·9H_2_O (19.69 mmol, 7.39 g) and benzene-1,3,5-tricarboxylic acid (BTC, C_9_H_6_O_6_) (6.32 mmol, 1.33 g) were mixed with 26.93 mL of deionised water inside a 125 mL Teflon container for around 40 min. After that, different amount of calcium carbonate (CaCO_3_) was added inside the above mixture according to the ratios given in Table S1.† Next, the Teflon container was capped and assembled into the steel case with tightly sealing and heated in a preheating oven at 493 K for 48 h. After that, the autoclave was left to cool at the laboratory temperature. Then, the vacuum filtration was done, and the crystalline collected product was washed thoroughly with deionised water. The splashed product was dried under vacuum at laboratory temperature and further dried at 373 K in an oven. The as-synthesised samples were activated by solvent exchange activation as described in the ESI.† This method is very active with using methanol as the exchangeable solvent.

### 2.3 Characterisation

Powder X-ray diffraction (XRD) measurements were taken using a Bruker D8 advance X-ray diffractometer with a Cu-Kα radiation source. A Fourier-transform infrared spectroscopy (FTIR) spectrometer (PerkinElmer) was used to find out Fourier transformer infrared spectra in a wave number ranged from 650 to 4000 cm^−1^. Thermal behaviour of the samples was determined by a thermogravimetric analysis (TGA) instrument (TGA/DSC1 STARe system-METTLER TOLEDO). The morphological description was achieved by using Zeiss Neon 40EsB FESEM. Elemental analysis of the samples was achieved *via* the elemental analysis instrument (ICP-OES). More characterisation details can be referred from the ESI section.†

### 2.4 Adsorption study

The CO_2_ (Laser Grade, 99.995%), He (UHP Grade, 99.999%) and N_2_ (UHP Grade, 99.999%) gases were supplied from BOC limited in Australia. Tristar-Plus-3020 and ASAP2050 (Micrometrics instruments, USA) were used for CO_2_ adsorption analysis at atmospheric pressure and high pressure respectively. While N_2_ adsorption was measured by ASAP2050 at 273 K and 290 kPa. An activated sample (0.09–0.15 g) was firstly dried in the oven for at least 1 h then the empty sample tube was weighed. After that, the dried sample was transferred into a weighed tube. Next, the filled tube was heated at 473 K under a vacuum for 8 h by a sample preparation system (VacPrep 061). Then, the net weight of the degassed sample was calculated. Finally, the degassed sample was analysed by the above instruments using a suitable analysing method. In addition, crushed ice was used to adjust the bath temperature at 273 K while the heating mantle of ASAP2050 was used to adjust other temperatures.

## 3. Results and discussions

### 3.1 Characterisations of MIL-96(Al) and a series of MIL-96(Al)–Ca samples

**Fig. 1:**
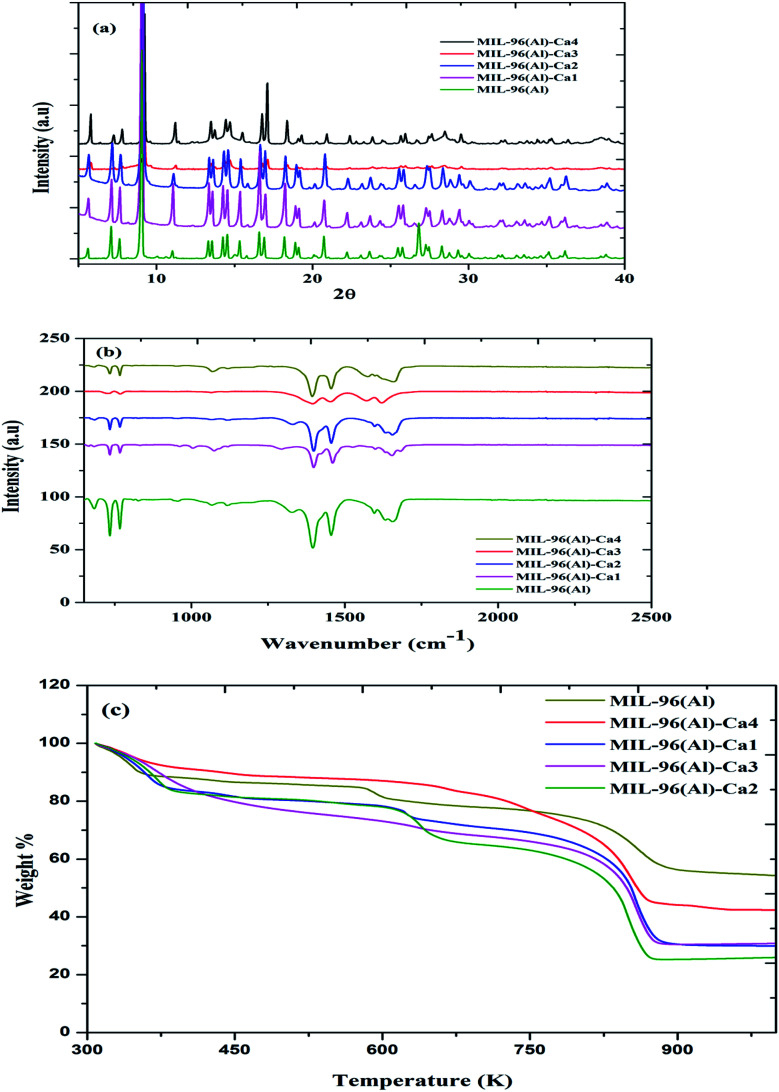
(a) XRPD profiles, (b) FTIR spectra, and (c) thermogravimetric analysis of MIL-96-Ca samples and MIL-96(Al).

Elemental analysis *via* the ICP instrument indicates that the four samples of MIL-96(Al)–Ca*N* (*N* = 1, 2, 3, 4) after activation process have the Ca^2+^/Al^3+^ mole ratios of 0.09, 0.32, 0.74 and 1.4%, respectively, as shown in Table S1.† The trace Ca content in the sample is possibly due to the Ca leaching out from the MOF structure during the methanol exchange activation process. The X-ray powder diffraction (XRPD) patterns of MIL-96(Al) and MIL-96(Al)–Ca samples are shown in Fig. 1a. The patterns demonstrate a high similarity in the MIL-96(Al) structure as reported previously.^42^ However, as the molar ratios of Ca^2+^/Al^3+^ in MIL-96(Al)–Ca3 and MIL-96(Al)–Ca4 increase, some defects are observed. As shown in the pattern of MIL-96(Al)–Ca3, the positions of the peaks overlap with the peaks in other samples but their intensity is significantly reduced, which is the prominent evidence of reducing the crystal size to nanometer range.^43^ Further justification could be that the second metal would cause the defects if it is incompatible with the main metal as these two metals have different coulombic charges and ionic sizes.^44^ FTIR spectra of MIL-96(Al)–Ca samples and MIL-96(Al) are shown in Fig. 1b. It can be seen that the infrared band at 1630 and 1332 cm^−1^ is allocated to the OH group bending in the plane of MIL-96(Al), MIL-96(Al)–Ca1 and MIL-96(Al)–Ca2. While this band disappears in the spectra of MIL-96(Al)–Ca3 and MIL-96(Al)–Ca4 of higher Ca^2+^ content. This observation might be attributed to the further interaction of OH^−^ bending with Ca^2+^ in the metal centre instead of its interaction with OH group of non-coordinated BTC molecules inside the pores.^45^ The spectrum of carboxylic groups in the BTC is usually seen in the spectral range from 1200 to 1700 cm^−1^. Specifically, protonated BTC restricted in the structure is detected at the band of 1684 cm^−1^ in the MIL-96(Al) and MIL-96(Al)–Ca1 while this is merged with the band of carboxylic group in the deprotonated BTC in other samples of higher Ca^2+^ content. Likewise, the vibrations of asymmetric and symmetric stretching COO of Al-carboxylate groups in the deprotonated BTC at the activated MIL-96(Al)–Ca and the parent sample are displayed in two pairs of the peaks. The first one is at 1660 and 1456 cm^−1^ as shown in the spectra of MIL-96(Al)–Ca except that in the spectrum of MIL-96(Al)–Ca3 the first peak in this pair is seen at 1624 cm^−1^; and the second pair is at 1598 and 1396 cm^−1^ in parent sample while these peaks shift to 1600 and 1400 cm^−1^ in MIL-96(Al)–Ca1 and MIL-96(Al)–Ca2; and shifts to 1567 and 1399 cm^−1^, and 1578 and 1395 cm^−1^ in MIL-96(Al)–Ca3 and MIL-96(Al)–Ca4 respectively.^41^ The spectrum of MIL-96(Al)–Ca3 is more affected because its crystal sizes are very fine. Consequently, the intensities of the peaks are reduced and shifted. Moreover, the peaks at 760 and 735 cm^−1^ are assigned to C–H out the plane of the BTC ring *via* the structures of the MOFs.^46^ The vibration band at 687 cm^−1^ is assigned to O–Al–O or O–(Ca) Al–O. This peak is attenuated as the Ca^2+^ content increased. It seems that the vibrational motion of Al centre is changed because Ca^2+^ occupies some active sites of aluminium cluster.

Thermal gravimetric analysis (TGA) profiles presented in Fig. 1c show that all samples have similar thermal stability. More specifically, the thermal profiles have exposed three steps of weight loss, the first step being around 373 K, which can be attributed to evaporation of the moisture during heating to the boiling point of water.^47^ The second step is caused by the burning of restricted molecules of the protonated BTC inside the pores. Also, this step might be affected by a coordinated Ca^2+^ in the metal centres, which is clearly seen at around 600 K in the parent sample as shown in the previous studies.^41^ As the Ca^2+^ content in the main structures increase, this step shifts to a higher temperature until it disappears in the thermal profile of MIL-96(Al)–Ca4 that confirms, higher content of Ca in the structure displaces the restricted free BTC out of the pores. Eventually, the third step represents the weight loss due to collapsing the whole structure at around 830 K because the connections between the linkers and the metal centres were broken.

**Fig. 2:**
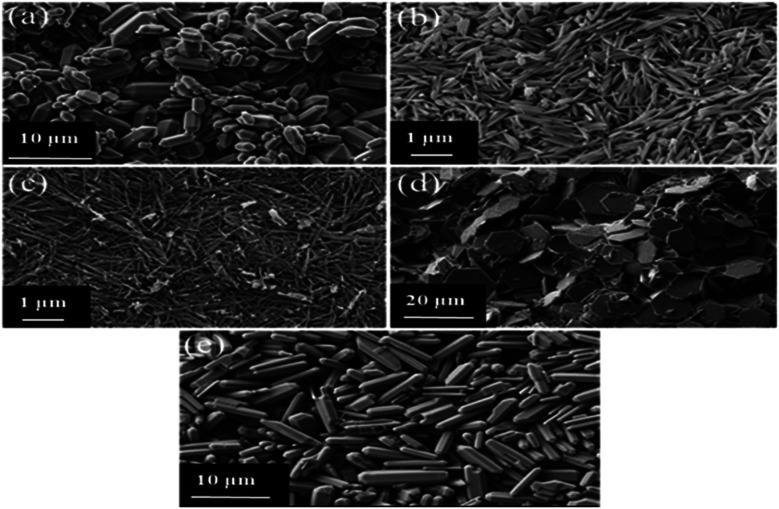
SEM images of (a) MIL-96(Al)–Ca1, (b) MIL-96(Al)–Ca2, (c) MIL-96(AL)–Ca3, (d) MIL-96(Al)–Ca4, and (e) MIL-96(Al).

Fig. 2a–e shows the morphological descriptions of MIL-96(Al)–Ca1, MIL-96(Al)–Ca2, MIL-96(Al)–Ca3, MIL-96(Al)–Ca4 and MIL-96(Al) sequentially. The Ca incorporation demonstrates an obvious effect in crystal size and morphology. Although the morphology of MIL-96(Al)–Ca1 in Fig. 2a shows a hexagonal rod capped by two hexagonal bipyramids likes what the parent sample (MIL-96(Al)) displays in Fig. 2e and the length of its crystals have significantly reduced. More specifically, crystals of the parent sample had lengths ranged from 5 to 12 μm, and diameters ranged from 2 to 5 μm whereas the lengths of the crystals of MIL-96(Al)–Ca1 appear much shorter (in the range of 2 to 8 μm). Moreover, the general morphological description of MIL-96(Al)–Ca2 remains as the morphology of the parent sample, whereas the diameter of the rod significantly reduces to 100 nm as shown in Fig. 2b. In addition, Fig. 2c shows that the shape of the crystals in MIL-96(Al)–Ca3 is a long rod with a very thin diameter (from 25 to 75 nm) lacking the hexagonal bipyramid on its ends. In contrast, Fig. 2d demonstrates a different morphology in MIL-96(Al)–Ca4 that is a thin hexagonal platelet (disk) with a diameter ranged from 10 to 20 μm and a thickness in a few nm. Remarkably, DSM can produce various morphological views and crystal sizes when the conditions of the synthesis are changed such as temperature, time and solvent.^48^ In this study, using Ca^2+^ as the second metal in the single pot synthesis caused the significant change in the morphology because it is directly affected the crystal anisotropy.^49^

**Fig. 3:**
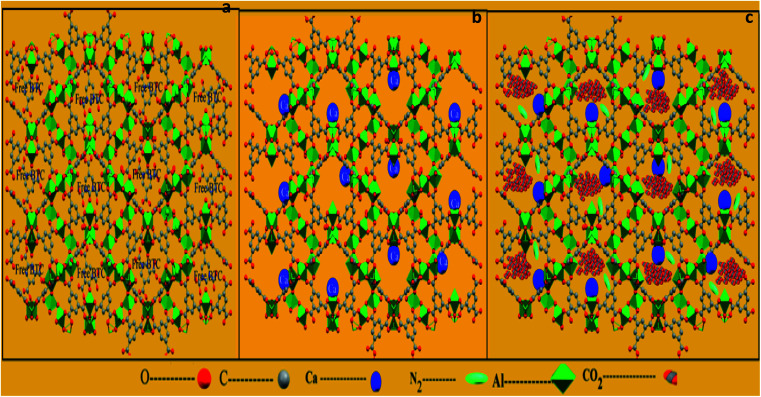
Framework of MIL-96-(Al) along the *a* axis (a); framework of MIL-96-(Al)–Ca (b) and framework of MIL-96-(Al)–Ca with CO_2_ or N_2_ capture (c).

This study confirms that the solvent exchange activation plays a main role in enhancing the textural properties by opening the metal sites of the prepared MIL-96(Al)–Ca samples. There are two reasons to justify this statement. One is the variety of the ionic sizes and valence numbers of Al^3+^ and Ca^2+^ and another is the efficiency of the activation process. Therefore, the second metal (Ca^2+^) does not replace the main metal (Al^3+^) as schematically shown in Fig. 3a and b, but it might coordinate with an active site of Al^3+^ metal centre (Fig. 3b). The methanol exchange activation is a very reliable method to leach out most coordinated Ca^2+^ by methanol leaving the opened metal sites for gas capture (Fig. 3c). However, the activated MIL-96(Al)–Ca samples still have traces of Ca^2+^ in the final products as presented in Table S1.†

### 3.2 CO_2_ and N_2_ adsorption behavior

**Fig. 4:**
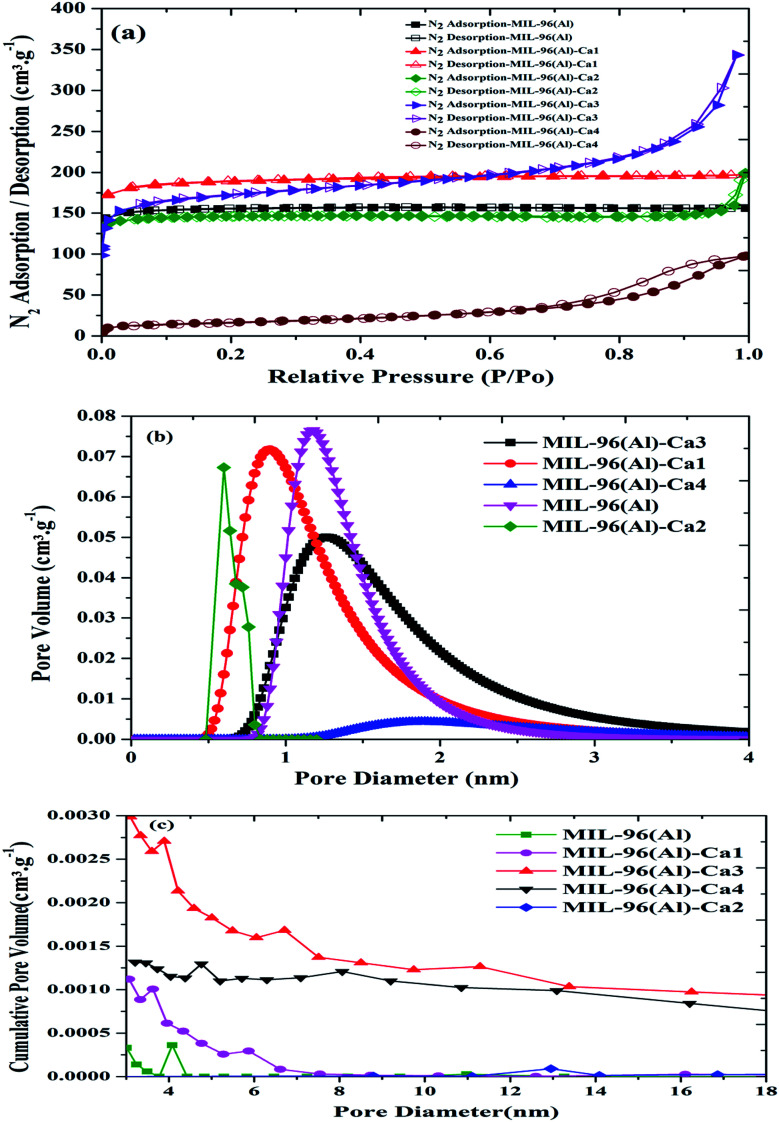
N_2_ adsorption/desorption isotherm (a), micropore distribution (b) and mesopore distribution (c) of MIL-96(Al)–Ca samples and MIL-96(Al).

The N_2_ adsorption/desorption isotherms at 77 K, micropore distribution and mesopore distribution of the MIL-96(Al)–Ca samples compared to the parent sample are shown in Fig. 4a–c. For each sample shown in Fig. 4a, a different hysteresis is obtained due to the presence of different pore sizes, including micropores and mesopores or their combined pores. Thus, it is expected that the slow desorption mechanism (percolation theory) might happen.^50^ Moreover, as shown in Table 1, the macropore content is remarkably increased as the content of Ca^2+^ is enhanced by more than 0.09%, and it is approved by a sharp increase of N_2_ adsorption when the pressure approached the atmospheric pressure. This is similar behaviour to that of MIL-100(Fe)–Ca in our previous work.^31^

**Table 1:** Textural properties of MIL-96-Ca samples and MIL-96(Al)

Adsorbents	*S*_BET_ (m^2^ g^−1^)	Average pore size (nm)	Pore volume (cm^3^ g^−1^)	Micropore content%
MIL-96(Al)	629.98	1.52	0.24	94.0
MIL-96(Al)–Ca1	754.57	1.60	0.30	92.3
MIL-96(Al)–Ca2	594.15	2.15	0.32	88.0
MIL-96(Al)–Ca3	660.26	3.21	0.53	76.5
MIL-96(Al)–Ca4	57.85	10.0	0.15	8.40

In addition, a significant change is observed in N_2_ adsorption of MIL-96(Al)–Ca4 which exhibits a larger hysteresis loop than other samples due to the majority of the mesopore and macropore in its structure. Fig. 4b shows the micropore distribution of MIL-96(Al)–Ca 1, 2, 3 and 4 compared to the parent sample. The availability of the smallest pore is seen in the samples of lower Ca^2+^ content, the pore diameter is 0.6 and 0.8 nm in MIL-96(Al)–Ca1 and MIL-96(Al)–Ca2 respectively. On the other hand, the micropore diameter in MIL-96(Al)–Ca3 remains at 1.4 nm in the parent sample and increases to 1.8 nm in MIL-96(Al)–Ca4.

The mesopore size distribution curves presented in Fig. 4c clearly show that both of the parent sample and MIL-96(Al)–Ca2 exposed very low cumulative volume (<0.0005 cm^3^ g^−1^) in a single unique peak of pore size centred at 4 and 13 nm respectively. MIL-96(Al)–Ca2 demonstrates the largest mesopore diameter with the lowest in the cumulative volume. Whereas in MIL-96(Al)–Ca1, the maximum cumulative volume is doubled (0.001 cm^3^ g^−1^) in smaller mesopore diameter of 3 nm, and that volume increases in MIL-96(Al)–Ca3 (0.0027 cm^3^ g^−1^) and MIL-96(Al)–Ca4 (0.0013 cm^3^ g^−1^) at mesopore diameter of 4 and 5 nm respectively. As a result, both population and the length of mesopore are significantly enhanced in the samples of the higher Ca^2+^ content.^51,52^

Table 1 illustrates the BET surface areas, average pore sizes, pore volumes, micropore contents. Recognisably, the BET surface area increases at the lowest loading of Ca^2+^ (0.09%) in MIL-96(Al)–Ca1, but it is declines at the highest Ca^2+^ content (1.4%) in MIL-96(Al)–Ca4. The BET surface areas are 629.98, 754.57, 594.15, 660.26 and 57.85 m^2^ g^−1^ in MIL-96(Al), MIL-96(Al)–Ca1, MIL-96(Al)–Ca2, MIL-96(Al)–Ca3 and MIL-96(Al)–Ca4, respectively while the average pore size in those samples are 1.52, 1.60, 2.15, 3.21 and 10 nm for the same order of the samples. The pore diameter in MIL-96(Al)–Ca4 is significantly higher than other samples. This can be interpreted by the expansion of a cage size, which is governed by the number of vertices and their connectivity. Therefore, the larger pore is possible to be dominated when the connectivity of vertices is reduced.^53^ It means when some of the active Al^3+^ sites are occupied by Ca^2+^, some of the vertices may lose some of their connectivity. Consequently, further enhancement in the pore size may occur when the Ca^2+^ are desolvated *via* the activation process. However, the pore volume is increased to 0.3, 0.32 and 0.53 cm^3^ g^−1^ in MIL-96(Al)–Ca1, MIL-96(Al)–Ca2 and MIL-96(Al)–Ca3 respectively, which is higher than the pore volume in MIL-96(Al) and reduced to 0.151 cm^3^ g^−1^ in MIL-96(Al)–Ca4 due to its lowest surface area. Finally, the microporosity is significantly decreased when the concentration of Ca^2+^ is raised to 1.4%. Hence, the micropore content is dropped from 94% in the parent sample to 8.4% in MIL-96(Al)–Ca4.

**Fig. 5:**
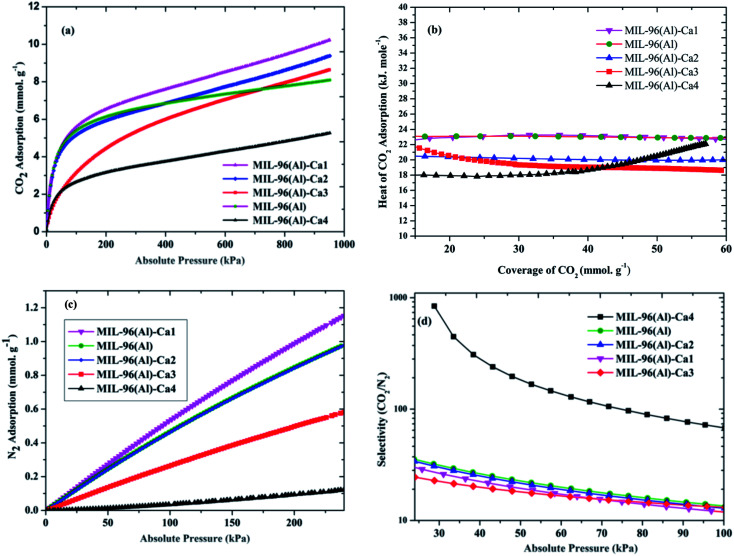
(a) CO_2_ adsorption at 273 K (b) heat of CO_2_ adsorption (c) N_2_ adsorption at 273 K, and (d) selectivity of CO_2_/N_2_ at STP in MIL-96(Al)–Ca samples and MIL-96(Al).

Fig. 5a shows the CO_2_ adsorption at 273 K and high pressure up to 950 kPa. It is noticed that the CO_2_ adsorption capacity is 8.09 mmol g^−1^ in the parent sample. Whereas this noticeably enhanced in MIL-96(Al)–Ca1, MIL-96(Al)–Ca2 and MIL-96(Al)–Ca3 as it is 10.22, 9.38 and 8.64 mmol g^−1^ respectively. However, CO_2_ adsorption decreases to about 5.26 mmol g^−1^ in MIL-96(Al)–Ca4 because further increasing in Ca^2+^ content leads to decrease in the BET surface area, pore-volume, micropore content.^54^ It seems that the pore volume expands when the high pressure is applied. Therefore, CO_2_ uptake is dramatically increased in the MIL-96(Al)–Ca samples with the Ca^2+^ content lower than 0.74%. Systematically, the CO_2_ adsorption in microporous materials increases when the pore volume and BET surface area are augmented and *vice versa*.^55^ It can be confirmed from Table 2 that the adsorption of CO_2_ is significantly boosted in most of the MOFs in this work and typically in MIL-96(Al)–Ca1 and MIL-96(Al)–Ca2. Although, almost all samples in the tabulated references have higher surface areas, they present lower CO_2_ adsorption capacity than the present work listed samples. Fig. 5b shows that the isosteric heat of CO_2_ adsorption changes depending on the CO_2_ coverages in MIL-96(Al)–Ca samples. The heat of adsorption is reduced with increasing the Ca^2+^ content. Generally, in the sample of the lowest Ca^2+^ content, the heat of adsorption slightly changes when the coverage of CO_2_ increases. More specifically, MIL-96(Al)–Ca1 and MIL-96(Al) have exposed similar heat of CO_2_ adsorption as it is 23 kJ mol^−1^ at CO_2_ coverage of 60 mmol g^−1^. In contrast, the heat of CO_2_ adsorption in MIL-96(Al)–Ca2 and MIL-96(Al)–Ca3 obviously decrease to 20 and 19 kJ mol^−1^ respectively. However, MIL-96(Al)–Ca4 shows a different behavior, the heat of adsorption is decreased further than that in MIL-96(Al)–Ca3 when the coverage of CO_2_ ranges from 10 to 38 mmol g^−1^, then it is increased over than both of MIL-96(Al)–Ca2 and MIL-96(Al)–Ca3 at a higher coverage of CO_2_. This heat of CO_2_ adsorption is 18 kJ mol^−1^ at CO_2_ coverage of 10 mmol g^−1^ and is 22 kJ mol^−1^ at CO_2_ coverage of 55 mmol g^−1^.

**Table 2:** The selectivity of the different adsorbents in comparison with MIL-96(Al)–Ca samples in this work

MOFs	Temperature (K)	Pressure (kPa)	CO_2_ adsorption capacity mmol g^−1^	Selectivity (CO_2_/N_2_) at 100 kPa	Specific surface area (m^2^ g^−1^)	Reference
Zeolite 13X	298	1000	6.5	7	—	61
Activated carbon	293	600	4.6	6.5	483	62
Activated carbon beads	293	1000	7.5	—	1457	63
MOF-5	297	1000	≈8	6	—	64
UiO-67	298	100	1.02	9.65	2505	65
UiO-66-NO_2_-(OH)_2_	298	900	5.6		732	66
Amino-UiO-66	298	500	5.5	31	1258	67
HKUST-1	305	500	1.75	—	1387.54	68
DUT-52	298	1000	6	—	1615	69
UiO-66(Zr)	293	1000	4.5	—	1125	70
MIL-53(Al)	303	1000	4.8	≈7.8	—	71
MOF-177	298	100	1.7	17	—	72
MOF-505	298	100	2.87	7.6	1104	73
In(iii)/Pd(ii)-MOF	273	100	4.1	18	795	74
MIL-101 (Cr, Mg)	298	100	3.25	—	3274	75
MOF 1-Co (Zn, Co)	273	100	2.45	—	3099	76
MIL-96(Al)	273	950	8.09	13.46	629.98	This work
MIL-96(Al)–Ca1	273	950	10.22	11.68	754.57	This work
MIL-96(Al)–Ca2	273	950	9.38	12	594.15	This work
MIL-96(Al)–Ca3	273	950	8.64	13.08	660.26	This work
MIL-96(Al)–Ca4	273	950	5.26	60.26	57.85	This work

The adsorption of CO_2_ and heat of adsorption are profoundly affected by functionalities and sizes of the pores. Furthermore, heterogeneity of the pores can be dominated in mesoporous materials.^56^ As a result, the homogenous adsorption can be anticipated to occur in the microporous adsorbents such as MIL-96(Al), MIL-96(Al)–Ca1 and MIL-96(Al)–Ca2 due to the homogeneity of the fine pores. Accordingly, the heat of CO_2_ adsorption is approximately uniform in those adsorbents.

The N_2_ adsorption capacities of MIL-96(Al)–Ca samples and the parent sample at extended pressure up to about 290 kPa are illustrated in Fig. 5c. Similarly, to the CO_2_ adsorption, the highest N_2_ adsorption is seen in MIL-96(Al)–Ca1. This material adsorbs 1.2 mmol g^−1^ of N_2_, which is higher than that of the parent sample and other MIL-96(Al)–Ca samples. Remarkably, N_2_ adsorption on MIL-96(Al)–Ca4 is very low (0.12 mmol g^−1^) due to the high average pore size.^57^ However, when the materials are ultrafine microporous as in the parent sample and MIL-96(Al)–Ca1 of smaller pores, the adsorption affinity of N_2_ molecules towards the surfaces of the pores is dramatically enhanced. In other words, the molecules of N_2_ can be actively interacted with the surface of the pores, as well as with each other due to the very limited space in the micropore.

In large pores, the interactions between the N_2_ molecules themselves are not likely to happen at normal temperatures. Also, in these conditions, the N_2_ molecules are weakly attached to the adsorption sites on the adsorbents because N_2_ molecules have a low quadrupole moment and low polarisability in addition to absence of the intensive electric field inside the large pores.^58^ Therefore, the adsorption capacities of N_2_ are extremely lower than those of CO_2_, which has much higher quadrupole moment and polarisability and lower kinetic diameter. The selectivity of CO_2_ over N_2_ at 100 kPa and 273 K is shown in Fig. 5d. In general, MIL-96(Al)–Ca4 presents significant separation factor among other samples in this study. The selectivity of CO_2_/N_2_ at 1 bar and 273 K is 13.46 in MIL-96(Al) while it is 11.68, 12, 13.08, and 60.26 in MIL-96(Al)–Ca1, MIL-96(Al)–Ca2, MIL-96(Al)–Ca3 and MIL-96(Al)–Ca4, respectively. In addition, when the pressure is far less than the atmospheric pressure, the selectivity is 80.53 (0.13 kPa), 113.15 (0.20 kPa), 77.51 (0.28 kPa), 53.60 (0.38 kPa) and 841.42 (28.80 kPa) in MIL-96(Al), MIL-96(Al)–Ca1, MIL-96(Al)–Ca2, MIL-96(Al)–Ca3 and MIL-96(Al)–Ca4, respectively. Notably, the selectivity on MIL-96(Al)–Ca4 at STP increases to around 5 times more than what is obtained on the parent sample and that reaches to about 26 times at 28.80 kPa.

It is clearly observed that a further increase in the pore size has significantly participated in enhancing the selectivity of CO_2_/N_2_ on MIL-96(Al)–Ca4. The improvement of this selectivity is because CO_2_ has higher polarisability, a higher quadrupole moment and a lower kinetic diameter. These parameters can highly enhance the adsorption of CO_2_ in the microporous and mesoporous materials with high superiority of adsorption in the ultrafine-microporous adsorbents.^59^ On the other hand, as it was mentioned above, the N_2_ molecule at STP conditions has very low affinity to be adsorbed in the porous materials with a majority of larger mesopore.^38,58^ However, although the microporous content, BET and CO_2_ adsorption is very low in MIL-96(Al)–Ca4, the affinity of this adsorbent for CO_2_ is much higher than that of N_2_ due to presence the Lewis base-calcium oxide coordinated on the metal center^60^. Thus, it produces a very high selectivity compared to other adsorbents reported in the literature as shown in Table 2.

## 4. Conclusions

Four samples of MIL-96(Al)–Ca and MIL-96(Al) were successfully synthesised, namely MIL-96(Al)–Ca1, MIL-96(Al)–Ca2, MIL-96(Al)–Ca3 and MIL-96(Al)–Ca4 with different Ca content. They were well characterised and used for CO_2_ and N_2_ adsorption. It was found that Ca^2+^ content in the final product is very low due to the incompatibility in the ionic size of Al^3+^ and Ca^2+^ and the high performance of the methanol exchange activation, but influencing the CO_2_ capture and selectivity significantly. The results reveal that CO_2_ adsorption is enhanced as the Ca^2+^ content increases up to 0.09% while it significantly dropped when the Ca^2+^ content reaches 1.4%. Moreover, the N_2_ adsorption sharply reduces when the Ca^2+^ content increases in MIL-96(Al)–Ca3 and MIL-96(Al)–Ca4. Consequently, the selectivity of CO_2_/N_2_ in MIL-96(Al)–Ca4 increases by a factor of 5 compared to MIL-96(Al). The present results suggest that MIL-96(Al)–Ca1 is a potential candidate for adsorption of CO_2_ while MIL-96(Al)–Ca4 is an excellent adsorbent for separation of CO_2_ from N_2_. These adsorbents may also have high potential usage for water treatment, deserving further investigations.

## Conflicts of interest

The authors declare no conflict of interest.

## Supplementary Material

RA-010-D0RA00305K-s001
