# Supplementary material for: Boosting CO2 adsorption and selectivity in metal–organic frameworks of MIL-96(Al) via second metal Ca coordination
Source: RSC Adv. 2020 Feb 25;10(14):8130–9. doi: 10.1039/d0ra00305k (PMC9049939; doi:10.1039/d0ra00305k)
Supplement: RA-010-D0RA00305K-s001 [file RA-010-D0RA00305K-s001.pdf]

Electronic Supplementary Material (ESI) for RSC Advances.
This journal is © The Royal Society of Chemistry 2020

Supporting Information

Boosting CO_2 Adsorption and Selectivity in Metal-Organic Framework of MIL-96 (Al) via the second metal-Ca coordination

Hussein Rasool Abid[a,b*], Zana Hassan Rada[a], Yuan Li[c,*], Hussein A. Mohammed[a], Yuan Wang[d], Shaobin Wang[e], Hamidreza Arandiyan[f], Xiaoyao Tan[c], Shaomin Liu[a,*]

[a] *WA School of Mines: Minerals, Energy and Chemical Engineering, Curtin University, WA 6102, Australia*

[b] *Environmental Department, Applied Medical Science, University of Karbala, Karbala 56001, Iraq*

[c] *Department of Chemical Engineering, Tianjin Polytechnic University, Tianjin, 300387, China*

[d] *School of Chemistry, Faculty of Science, The University of New South Wales, Sydney, New South Wales 2052, Australia*

[e] *School of Chemical Engineering, University of Adelaide, SA 5005, Australia*

[f] *Laboratory of Advanced Catalysis for Sustainability, School of Chemistry, The University of Sydney, Sydney 2006, Australia*

**Corresponding authors E-mail address:* hussein.abid@curtin.edu.au (H. Abid);

liyuan@tjpu.edu.cn (Y. Li);

Shaomin.Liu@curtin.edu.au (S.Liu)

Synthesis Method

During the synthesis procedure of MIL-96(Al)-Ca samples, the specific weight of Ca was lastly added to the synthesis procedure of MIL-96(Al) in a relative to the constant moles of Al as shown in below Table S1.

Table S1 The Molar Ratios of Ca^{+2}/Al^{+3} in the single pot synthesis procedure and the final Ca content in the activated sample.

Adsorbent	$CaCO_3$ (g)	Ca^{+2}/Al^{+3} (mol/mol) Direct synthesis	Ca^{+2}/Al^{+3} (mole/mole) % Activated sample
MIL-96(Al)	0	0	0
MIL-96(Al)-Ca1	0.15	0.076	0.09
MIL-96(Al)-Ca2	1.0	0.507	0.32
MIL-96(Al)-Ca3	2.0	1.01	0.74
MIL-96(Al)-Ca4	3.0	1.52	1.4

Activation of MIL-96(Al)-Ca Samples

Solvent exchange activation was used to discard the impurities and non-reacted precursors out the pores, with exchanging the second metal by the solvent molecules.

In simple steps, MIL-96(Al)-Ca samples were activated by immersing the dried products into methanol for 5 d then after that, it was filtered and dried by the vacuum filtration. Eventually, the final product was heated under vacuum at 473 K for 12 h. As a matter of fact, the heating temperatures is lower than the decomposition temperatures in the TGA profiles to ensure the integrity of the structures after the activation process.

Characterisation Methods

D8 Advance (Bruker AXS, Germany) was used to investigate X-Ray powder diffraction (XRPD) patterns. The activated powders were packed in the normal holders and then scanned in a range of 2-theta from 5 to 60 degree. Also, the textural properties (the specific surface area, pore size and pore volume) of adsorbents were revealed by measuring N_2 adsorption-desorption isotherms, which were obtained at 77 K by Autosorb1 instrument. Weight of a sample ranged from 0.09 to 0.15 g was firstly dried in an oven then poured in a sample tube which was assembled to the degassing station at 473 K for a period until the degassing process was completed. Finally, the net weight of the sample was calculated, and the sample tube was transferred to the analysis station for further analysis after setting a suitable analysing method.

.

Thermal stability and weight loss profiles of the samples were determined by a thermogravimetric analysis (TGA) instrument (TGA/DSC1 STARe system-METTLER TOLEDO). A 15 mg of a sample was loaded into an alumina pan then heated in the TGA furnace in the air environment at a rate of 10 K/min from 303 to 1173 K.

The morphological description was achieved by using Zeiss Neon 40EsB FESEM. Firstly, a 5 mg of a sample was suspended in a 5 mL of absolute ethanol then a carbon conductive tab was stuck on the top surface of the SEM specimen stub. After that, the suspension was dropped on the carbon tab. Then, it was left for enough time to be totally dried. Next, it was coated by 2μ of Platinum and finally used in the instrument for an SEM test.

Elemental analysis

Prior to using the elemental analysis instrument (ICO-OES), the solid samples must be transferred to the liquid phase by a process called digestion. Final phase of the sample must be clear liquid. There are many standard methods used for the digestion. These methods are

usually used strong acids such as HF, HNO_3, HCl, H_2SO_4 and H_3PO_4. The selection of digesting acid is based on the type of metals presented in the sample. As a result, some metals need one acid to be precisely digested and others might need mixed acids.

Also, it is preferred to do the digestion in a suitable container which not be attacked by the acid. For more accurate results, the acid digestions should be achieved in the closed vessels such as a pressure glass vial or a PTFE high-pressure acid digestion vessel cupped and covered in a strong outer stainless steel body [1, 2]. Activated MIL-196(Al)-Ca samples were not digested in the closed glass vial, therefore, PTFE -Teflon lined autoclave was used. Firstly, 10 mg of a sample was treated with 10 mL of nitric acid (72%) and 2 mL of hydrochloric acid (32%). Then, the mixture was transferred into Teflon-line autoclave of 45 mL, which was tightly sealed and heated at 473 K for 12 h. Eventually, after cooling, the digested mixture was filtered by a 0.45 μm syringe filter and diluted 36 times to get the suitable concentration for both of the acids, then it was used for analysis by ICP-OES to get the results as displayed above in Table S1.

Heat of Adsorption

Isosteric heat of adsorption was calculated using the Clausius–Clapeyron equation [3, 4].

$$ln\left[\frac{P}{P_o}\right] = \frac{\bar{h}(n)}{R}\left[\frac{1}{T} - \frac{1}{T_o}\right]$$ (When n is constant)--------------------- (Eq. S1)

Where R is the universal gas constant, P is the absolute pressure of a gas at T (Temperature) while P_o is the absolute pressure of the gas at T_o. The $\bar{h}$(n) is the isosteric heat of adsorption at n where n is the amount of adsorbed gas. The heat of CO_2 adsorption on MIL-96(Al)-Ca samples was calculated by eq. S1 using the CO_2 adsorption at 273 and 298K in Figure S1 (a-e).

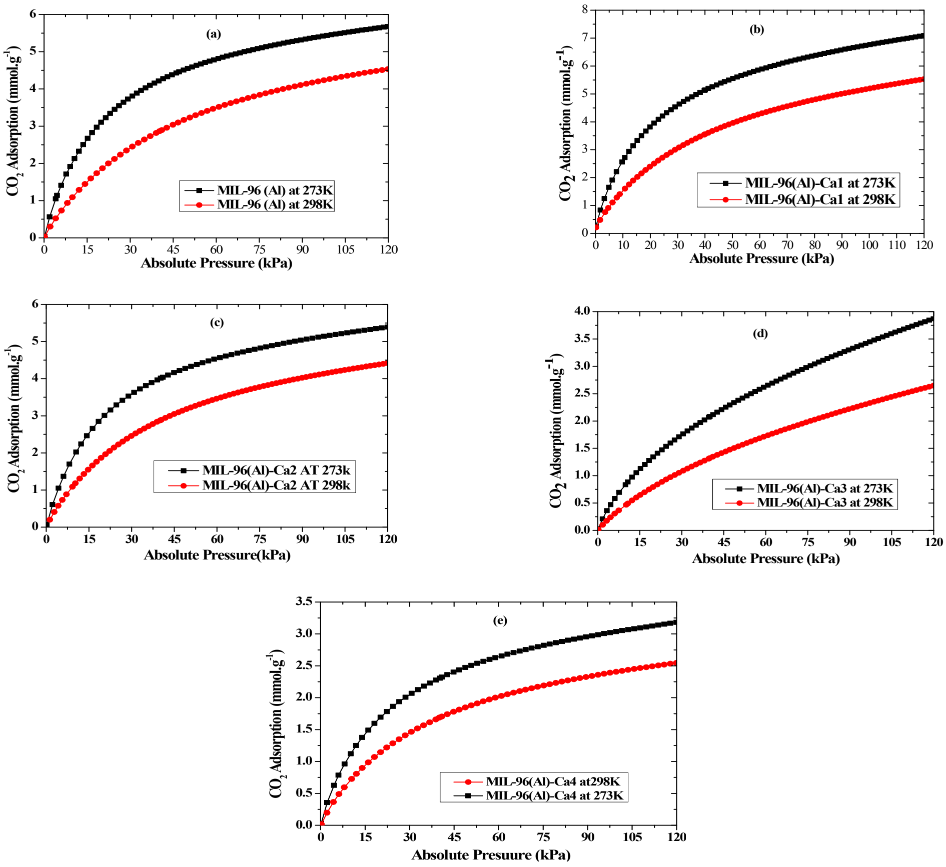

Figure S1 (a, b, c, d and e) CO_2 adsorption on MIL-96(Al)-Ca samples at atmospheric pressure and different temperatures.

Selectivity (CO_2/N_2)

The selectivity was calculated using the isotherms of the single gas adsorption of CO_2 and N_2 at the same conditions as described in the following equation;

$$selectivity\frac{CO_2}{N_2} = \left[\frac{V_{CO_2}}{V_{N_2}}\right]_{(T,P)}$$ --- (Eq. S2)

Where V_{CO2} and V_{N2} indicates the volumes of gases CO_2 and N_2 respectively at T and P [5].

References

1. J. S. F. Pereira, C. L. Knorr, L. S. F. Pereira, D. P. Moraes, J. N. G. Paniz, E. M. M. Flores and G. Knapp, *Journal of Analytical Atomic Spectrometry*, 2011, **26**, 1849-1857.
2. A. Gonzálvez, S. Armenta, A. Pastor and M. de la Guardia, *Journal of Agricultural and Food Chemistry*, 2008, **56**, 4943-4954.
3. S. Builes, S. I. Sandler and R. Xiong, *Langmuir*, 2013, **29**, 10416-10422.
4. A. L. Myers, *AIChE Journal*, 2002, **48**, 145-160.
5. Z. H. Rada, H. R. Abid, H. Sun, J. Shang, J. Li, Y. He, S. Liu and S. Wang, *Progress in Natural Science: Materials International*, 2018, **28**, 160-167.
